# Deceased kidney donor cystatin C and subsequent recipient measured glomerular filtration rate at one year after transplantation

**DOI:** 10.1371/journal.pone.0342497

**Published:** 2026-03-10

**Authors:** Isabelle J. C. Dielwart, Daan Kremer, Tim J. Knobbe, Dion Groothof, Henri G. D. Leuvenink, Jenny E. Kootstra-Ros, Martin H. de Borst, Marco van Londen, Jan-Stephan F. Sanders, Robert A. Pol, Stephan J. L. Bakker, TransplantLines investigators

**Affiliations:** 1 Department of Internal Medicine, Division of Nephrology, University of Groningen, University Medical Center Groningen, Groningen, The Netherlands; 2 Department of Surgery, University of Groningen, University Medical Center Groningen, Groningen, The Netherlands; 3 Department of Laboratory Medicine, University of Groningen, University Medical Center Groningen, Groningen, The Netherlands; Instituto do Cancer do Estado de Sao Paulo, BRAZIL

## Abstract

**Background:**

Deceased donor kidney selection is largely determined by creatinine-based kidney function estimation. However, muscle wasting is common in potential donors and affects the accuracy of creatinine-based kidney function assessment. Cystatin C could serve as a muscle-mass independent alternative for this purpose. The primary aim of this study was to evaluate the associations of donor creatinine and cystatin C with recipient measured glomerular filtration (mGFR) at one year after transplantation.

**Methods:**

Using data from the prospective TransplantLines study, multivariable linear regression analyses were performed to examine the associations of donor plasma creatinine and cystatin C with recipient I^125^-iothalamate mGFR.

**Results:**

Donor plasma creatinine and cystatin C data were available for 96 donor-recipient pairs. Median pre-donation creatinine and cystatin C concentrations were 56.0 [49.5–71.0] µmol/L and 0.63 [0.50–0.82] mg/L, respectively. Recipient mGFR data at one year after transplantation were available in 55 kidney transplant recipients. Mean recipient mGFR was 52.1 ± 17.0 ml/min. Donor plasma creatinine was not associated with recipient mGFR (st. β −0.19, 95% CI [−0.48 to 0.10], p = 0.21), while donor cystatin C was significantly associated with recipient graft function (st. β −0.52, [−0.83 to −0.21], p = 0.002). The association of cystatin C and recipient mGFR remained materially unchanged after adjustment for potential confounders.

**Conclusion:**

In deceased kidney donors, donor plasma creatinine is not associated with recipient mGFR, whereas donor cystatin is associated. Addition of cystatin C to the assessment of deceased kidney donors may provide additional information in difficult cases and improve the accuracy of deceased kidney donor selection.

## Introduction

Kidney transplantation is the optimal treatment for kidney failure in most patients. Unfortunately, there is a large gap between the numbers of patients waiting and those receiving a transplant [1]. To increase the donor pool, traditional donor criteria have been expanded. As a result, kidneys are now accepted from donors who were previously considered suboptimal, including those classified as donation-after-circulatory death (DCD) and extended criteria kidney donors (ECD) [2,3]. The definition of ECD varies across countries but typically refers to older donors or those with health conditions that may increase the risk of primary non-function, graft dysfunction, and subsequent graft failure [2]. There is considerable variability among transplant nephrologists in deciding whether to accept kidneys from suboptimal donors [3–5]. This variability contributes to significant differences in discard rates across transplant programs, potentially leading to the rejection of suitable kidneys and acceptance of marginal ones [6–9].

The decision to accept a deceased kidney donor offer largely depends on a combination of donor related risk factors, including donor age, sex, type (donation after brain death (DBD) or donation after circulatory death (DCD)) and a renal biomarker reflecting kidney function of the donor. Within the Eurotransplant kidney allocation system, this renal biomarker is plasma creatinine [10]. While plasma creatinine often provides an adequate estimate of kidney function in the general population, challenges arise in populations where muscle mass deviates substantially from average, such as elderly deceased donors with high comorbidity rates [11–13]. Also during critical illness, muscle mass can decrease up to 1–2% per day, leading to a further decrease in muscle mass in patients admitted to intensive care units, and this includes potential deceased kidney donors [14,15]. Given that 98% of circulating creatinine stems from the spontaneous, nonenzymatic conversion of creatine into creatinine [16], decreased muscle mass results in overestimation of GFR [14]. Due to this overestimation, the actual GFR of deceased donors may be considerably lower than expected based on the pre-donation creatinine concentrations. Therefore, there is an urgent clinical need for muscle-mass independent biomarkers that can more accurately predict future recipient graft function.

Cystatin C is produced by all nucleated cells and therefore independent of muscle mass [17]. This property makes it a promising alternative to creatinine for GFR assessment in deceased kidney donors [12]. However, the association between cystatin C and future recipient graft function remains unclear. The primary aim of this study was to investigate the associations of donor plasma creatinine and donor plasma cystatin C with recipient measured GFR at one year after kidney transplantation. Secondary, we aimed to assess the association of donor creatinine and cystatin C with recipient 24-h creatinine clearance.

## Methods

This study was conducted and reported following the Strengthening the Reporting of Observational studies in Epidemiology (STROBE) Guidelines [18]. The completed checklist is available in S1 Fig.

### Study population

All deceased kidney donors, who donated at least one kidney to a recipient transplanted in the University Medical Center Groningen (UMCG), Groningen, the Netherlands, from 01/01/2004 until 31/12/2020, were included in this study. Recipients were excluded if no data on measured GFR or creatinine clearance at one year after transplantation was available. Additionally, we excluded all donors with acute kidney injury, defined as values exceeding two standard deviations above the mean of donor pre-donation creatinine concentration. Donor written consent was obtained from their relatives, according to the applicable rules and agreements of the Dutch Transplant Foundation (NTS) [19]. In accordance with Dutch legislation prior to 2018, general consent for transplantation included permission for participation in research, without need for written informed consent. For all recipients enrolled after 2018, written informed consent was obtained within the framework of the ongoing, prospective TransplantLines Biobank and Cohort study (Clinical Trails.gov identifier: NCT03272841). All procedures were carried out in accordance with the protocol approved by the Committee of the UMCG (METc number 2014/077) and adheres to the Declaration of Helsinki and Istanbul.

### Data collection

All donor data were available through the TransplantLines Biobank and Cohort Study [19]. Additional recipient information was collected from electronic patient files between 01/08/2022 to 01/12/2022. After data collection, the data was stored under pseudonymized identifiers. All data handling complied with institutional privacy regulations.

Donor blood samples were prospectively collected prior to the organ procurement procedure. All collected samples were centrifuged at 2800G at 4˚C for ten minutes and subsequently stored at −80˚C until analyses were performed. Recipient blood sample and 24-h urine samples were collected before the study visit. Plasma and urinary creatinine concentration were routinely measured using an enzymatic assay (Roche Diagnostics, Basel, Switzerland). Cystatin C levels were measured in EDTA plasma using a validated particle-enhanced turbidimetric immunoassay. Recipient GFR (mGFR) was measured using ^125^I-iothalamate and ^131^I-hippurate infusion at one year after transplantation [20].

### Statistical analysis

Descriptive data were generated for all variables. Baseline characteristics are presented as mean ±SD for normally distributed data, as median [Q1-Q3] for non-normally distributed variables and as number (valid %) for categorical data. Data distribution was visually assessed using histograms and Q-Q plots. First, we assessed the association of donor creatinine and cystatin C with recipient mGFR at one year after transplantation, using univariable linear regression analyses. Adjustment was performed for donor age and sex (Model 1). To maintain the number of variables proportional to the number of cases, further adjustments were additive to Model 1 in the following manner: (i) donor type in Model 2, (ii) donor BMI in Model 3, (iii) donor history of smoking, hypertension and diabetes mellitus in Model 4, (iv) recipient age and sex in Model 5. Next, we assessed the association of donor creatinine and cystatin C with recipient 24-h creatinine clearance using linear regression analyses with the same mentioned models. In sensitivity analyses, we assessed the association of donor creatinine and cystatin C with recipient mGFR indexed to body surface area (BSA) and urinary creatinine clearance indexed to BSA. In case of missing cases in covariables pairwise exclusion was used. Finally, we assessed potential effect modification by adding interaction terms with donor age, sex and type in all models. Statistical analyses were performed in SPSS version 23 for windows and R studio version 4.3.1. Claude.ai was used to support R code development for data analyses and improve the quality and coherence of the text. A P-values <0.05 was considered to indicate statistical significance.

## Results

### Study population

**Fig 1:**
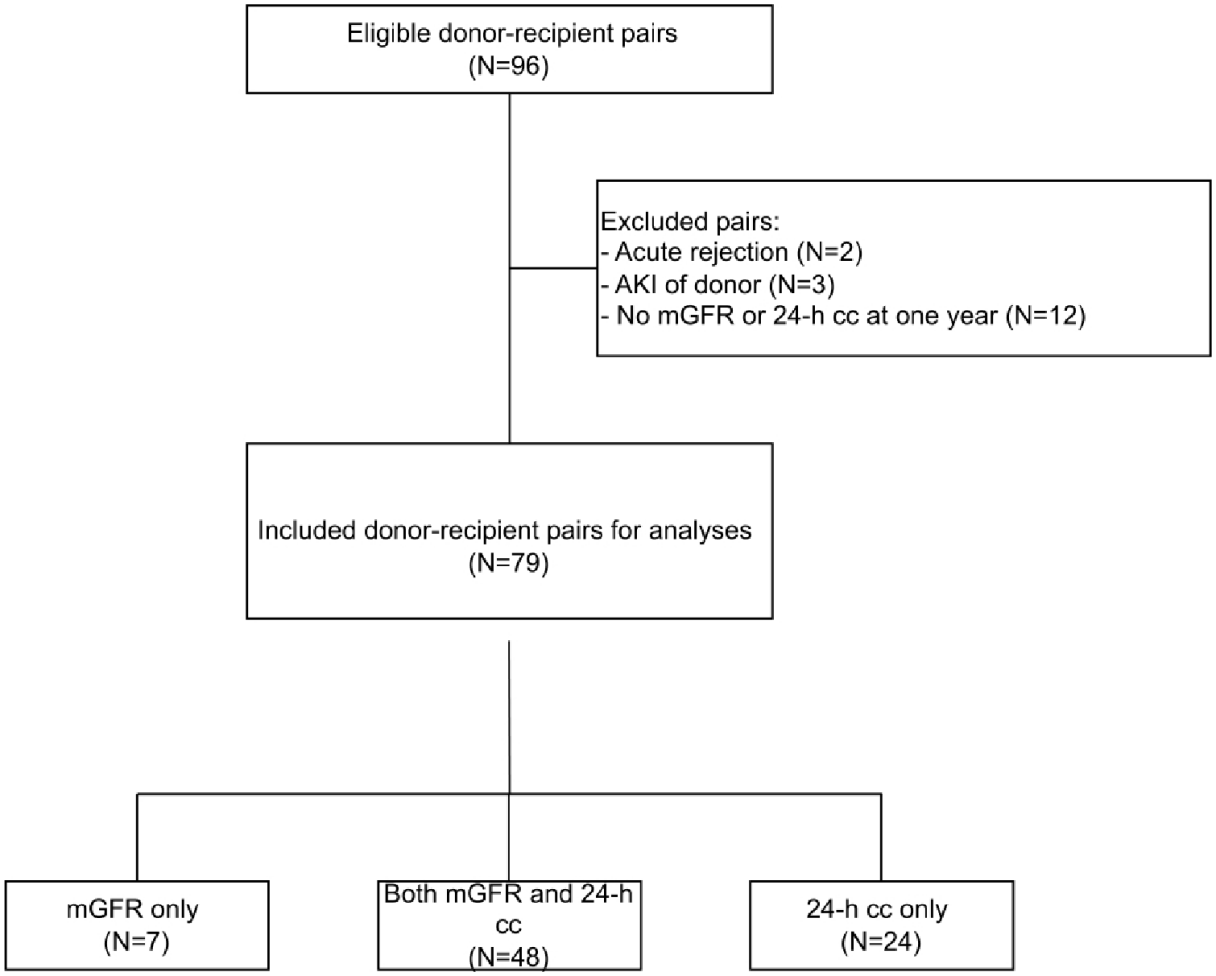
Flowchart.

Donor cystatin C data were available for 96 donor-recipient pairs, of which 12 (12.5%) were excluded because of the absence of data on recipient mGFR or 24-h creatinine clearance. Another 2 (2.0%) recipients were excluded from analyses because they experienced graft failure based on acute rejection. Additionally, 3 (3.1%) donor-recipient pairs were excluded because of acute kidney injury of the donor. Consequently, a total of 79 donor-recipients pairs were eligible for analyses. Data on both mGFR and 24-hour creatinine clearance were available for 48 (60.8%) recipients, while mGFR alone was available for 7 (8.9%) recipients and 24-hour creatinine clearance alone was available for 24 (30.3%) recipients (Fig 1). Among the donors, 38 (48.1%) were female, mean age was 49.3 ± 17.8 years and 40 (50.6%) donated after circulatory death (DCD). Median pre-donation creatinine and cystatin C concentrations were 56.0 [49.5–71.0] µmol/L and 0.63 [0.50–0.82] mg/L respectively. Among recipients, 35 (44.3%) were female, mean age at transplantation was 57.1 ± 12.3 years and mean mGFR and 24-h creatinine clearance were 52.1 ± 17.0 ml/min and 57.7 ± 24.0 ml/min respectively. Population characteristics of both donors and recipients are presented in Table 1.

**Table 1:** Population characteristics.

	Donor (n = 79)	Recipient (n = 79)
Female sex, *n (%)*	38 (48.1%)	35 (44.3%)
Age, years	49.3 ± 17.8	57.1 ± 12.3
BMI, kg/m^2^	25.3 ± 4.5	26.7 ± 5.0
Donation after circulatory death (DCD), *n (%)*	40 (50.6%)	
History of smoking, *n (%)*	32 (40.5%)	
History of hypertension, *n (%)*	13 (16.5%)	
History of diabetes mellitus, *n (%)*	5 (6.3%)	
Pre-donation creatinine concentration, µmol/L	56.0 [49.5 to 71.0]	
Pre-donation cystatin C concentration, mg/L	0.63 [0.50 to 0.82]	
Recipient creatinine concentration, µmol/L		138 [102.5 to 166.5]

Population characteristics. Categorical variables are presented as n(%), normally distributed continuous variables as mean (SD) and non-normally distributed continuous variables as median [IQR]. Data regarding 12-months creatinine concentration were missing for 5 recipients.

### Primary analyses

#### Pre-donation creatinine, cystatin C and recipient mGFR.

**Fig 2:**
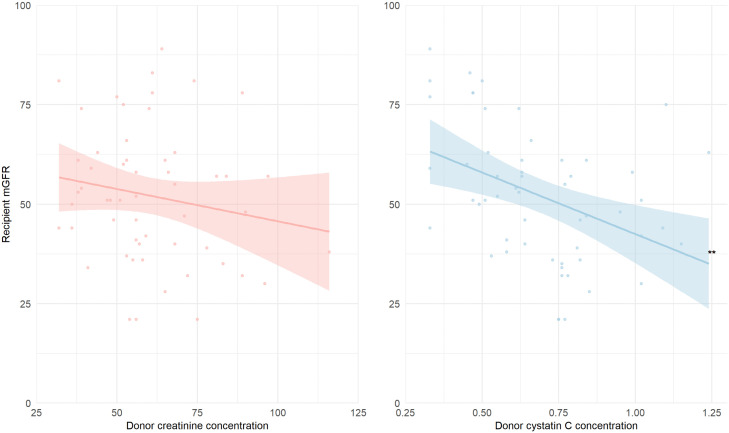
Association of donor creatinine, cystatin C with recipient mGFR. **p < 0.01. Associations of donor creatinine- (red) and cystatin C concentration (blue) and recipient mGFR. The light area represents the 95% CI of the regression line. Data on recipient mGFR available of 55 kidney transplant recipients.

Higher pre-donation creatinine was not significantly associated with recipient mGFR at one year after transplantation (standardized (st.) β −0.19, 95% CI [−0.48 to 0.10], and p = 0.21), while higher donor cystatin C concentration was significantly associated with lower recipient mGFR (st. β −0.52 [−0.83 to −0.21], p = 0.002), as visualized in Fig 2. The latter association was independent of potential confounders, including donor age and sex (Table 2).

**Table 2:** Association of donor creatinine, cystatin C with recipient mGFR.

Outcome: recipient mGFR								
	*Creatinine*				*Cystatin C*			
	st.ß	95% CI	R^2^	p-value	st.ß	95% CI	R^2^	p-value
Univariable	−0.19	[-0.48 to 0.10]	0.01	0.21	−0.52	[-0.83 to -0.21]	0.15	0.002
Model 1	−0.08	[-0.36 to 0.20]	0.31	0.57	−0.41	[-0.69 to -0.13]	0.41	0.006
Model 2	−0.08	[-0.36 to 0.21]	0.31	0.60	−0.41	[-0.69 to -0.12]	0.40	0.007
Model 3	−0.11	[-0.41 to 0.18]	0.31	0.45	−0.44	[-0.73 to -0.16]	0.41	0.003
Model 4	−0.09	[-0.39 to 0.21]	0.30	0.56	−0.42	[-0.72 to -0.12]	0.39	0.009
Model 5	−0.12	[-0.40 to 0.16]	0.34	0.40	−0.42	[-0.69 to -0.14]	0.43	0.005

Recipient mGFR data were available for 55 (69.6%) recipients.

There was no missing data in the covariables for the primary analyses.

Model 1: adjustment for donor age and sex. Model 2: donor age, sex and donor type. Model 3: donor age, sex and BMI. Model 4: donor age, sex, history of hypertension, smoking and diabetes mellitus. Model 5: donor age, sex and recipient age and sex.

### Secondary analyses

#### Donor creatinine, cystatin C and recipient 24-h creatinine clearance.

**Fig 3:**
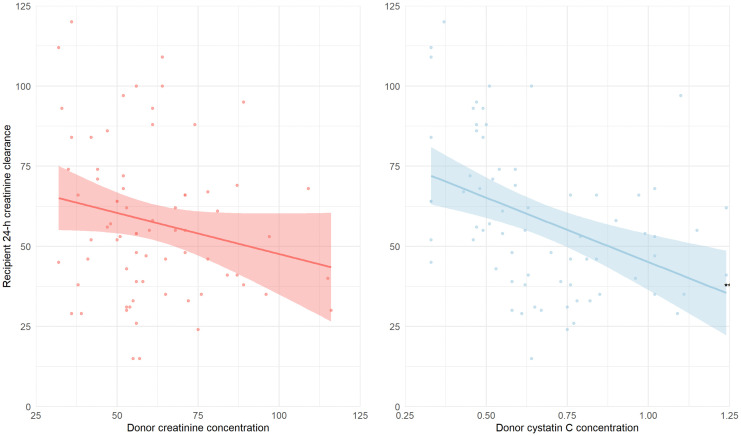
Association of donor creatinine, cystatin C and recipient 24-h creatinine clearance. *** p < 0.001. Associations of donor creatinine- (red) and cystatin C concentration (blue) and recipient 24-h creatinine clearance. The light area represents the 95% CI of the regression line. Data on recipient 24-h creatinine clearance available of 72 kidney transplant recipients.

Data on recipient 24-h creatinine clearance were available for 72 recipients (91%). Similar to the results of associations with mGFR, higher pre-donation creatinine was not significantly associated with 24-h creatinine clearance (st. β −0.21 [−0.43 to 0.02], p = 0.08), while pre-donation cystatin C was (st. β −0.42 [−0.62 to −0.22], p < 0.001) (Fig 3 and Table 3).

**Table 3:** Association of donor creatinine, cystatin C and recipient 24-h creatinine clearance.

Outcome: 24-h creatinine clearance								
	Creatinine				Cystatin C			
	st.ß	95% CI	R^2^	p-value	st.ß	95% CI	R^2^	p-value
Univariable	−0.21	[-0.45 to 0.02]	0.03	0.08	−0.42	[-0.62 to -0.22]	0.19	<0.001
Model 1	−0.26	[-0.51 to -0.01]	0.16	0.04	−0.37	[-0.57 to -0.17]	0.26	<0.001
Model 2	−0.27	[-0.52 to -0.01]	0.15	0.04	−0.38	[-0.58 to -0.17]	0.25	<0.001
Model 3	−0.26	[-0.52 to -0.01]	0.15	0.05	−0.37	[-0.58 to -0.17]	0.25	<0.001
Model 4	−0.29	[-0.55 to -0.03]	0.16	0.03	−0.38	[-0.59 to -0.17]	0.24	<0.001
Model 5	−0.3	[-0.55 to -0.05]	0.21	0.02	−0.4	[-0.59 to -0.21]	0.31	<0.001

Recipient 24h-creatinine clearance data were available for 72 (91.1%) recipients.

There was no missing data in the covariables for the primary analyses.

Model 1: adjustment for donor age and sex. Model 2: donor age, sex and donor type. Model 3: donor age, sex and BMI. Model 4: donor age, sex, history of hypertension, smoking and diabetes mellitus. Model 5: donor age, sex and recipient age and sex.

### Sensitivity analyses

#### Associations of donor creatinine and cystatin C with recipient BSA-indexed mGFR and creatinine clearance.

Mean recipient BSA-indexed mGFR and 24-h creatinine clearance were 47.5 ± 14.9 ml/min/1.73m^2^ and 51.6 ± 20.4 ml/min/1.73m^2^ respectively. Donor cystatin C was negatively associated with recipient BSA-indexed mGFR (st. β −0.36 [−0.70 to −0.02], p = 0.04). This association was lost after adjustment for donor age and sex (p = 0.19) (S1 Table). Results of analyses with recipient creatinine clearance indexed for BSA were comparable to those with 24-h creatinine clearance (S1 Table).

## Discussion

In this study, we showed that donor creatinine was not associated with recipient mGFR and 24-h creatinine, while cystatin C was significantly associated with recipient outcomes. The latter association remained significant after adjustment for potential confounders. These findings suggest that the current practice of relying solely on donor creatinine for donor GFR assessment can benefit from adding cystatin C. Sole reliance on creatinine could result in the exclusion or misclassification of potentially suitable kidney donors.

Our findings demonstrate significant limitations in donor creatinine as marker of glomerular filtration rate and this becomes particularly problematic in the context of deceased donors as circulating creatinine is influenced by non-renal factors, like muscle mass. Deceased kidney donors, particularly those meeting extended criteria, are likely to have muscle mass that deviates significantly from the general population, due to older age and higher comorbidity rates [11,21]. In addition, due to critical illness, donors lose approximately 1–2% of muscle mass per day, resulting in a falsely elevated estimated GFR of 2 ml/min/1.73m^2^ per day which can further compromise donor selection processes [14,22,23].

While GFR measurement using an external marker (iothalamate, iohexol) remains the gold standard for kidney function evaluation, logical constraints make it impractical for the deceased donor population to incorporate [20]. Instead, donor GFR assessment using creatinine and cystatin C, in addition to other donor-specific risk factors may provide a more robust evaluation [24]. This approach could be particularly beneficial for the assessment of potential kidney donors who have experienced prolonged ICU stays, as creatinine has been shown to underperform in the assessment of GFR in the critically ill population, whereas cystatin C demonstrates superior performance in this setting [14,25].

Despite its potential utility, the use of cystatin C in deceased donor screening may be limited by other non-GFR-related confounding factors specific to critically ill patients, like deceased kidney donors. These factors include smoking, corticosteroid exposure and inflammation [26–28]. In experimental models, an inflammatory response following severe brain injury has been shown to increase cystatin C expression in rats [29,30]. Consequently, the pathophysiology of brain death and its associated treatment with corticosteroids may affect circulating cystatin C levels particularly in donation after brain death (DBD) kidney donors. In addition, critically ill patients often exhibit substantial variability in circulating cystatin C levels, which complicates its interpretation [31]. However, adjustment for donor type (DBD vs DCD), BMI, and clinically relevant covariates, including smoking status and diabetes did not materially alter our findings, indicating that these non-GFR determinants are unlikely to fully explain the observed association between donor cystatin C and recipient mGFR.

Values of measurements of plasma or serum creatinine and plasma or serum cystatin C are frequently incorporated separately or combined in formula’s to estimate the GFR (eGFR) [32]. However, when assessing the quality of a deceased kidney donor offer several other donor-related risk factors are considered alongside of plasma or serum creatinine (e.g., donor age, donor sex, donor type and cause of death) [33]. Within the Eurotransplant allocation system, values of plasma or serum creatinine, rather than eGFR, are principally reported as renal biomarker [10]. Similarly, in the United States, the kidney donor profile index (KDPI) is reported, which is a score that informs clinicians about the quality of a deceased donor kidney [32]. This score includes values of plasma or serum creatinine, rather than eGFR [32]. Therefore in this study we aimed to identify the individual associations of both plasma creatinine and plasma cystatin C with recipient mGFR, while adjusting for potential confounders. We showed that donor plasma cystatin C was significantly and independently associated with recipient mGFR, while this was not true for donor plasma creatinine.

Among all assessed potential confounding variables, only increasing donor age was significantly associated with lower recipient mGFR and 24-h creatinine clearance. Donor age is a well-known determinant of kidney transplantation outcomes [2,34–36], and its strong correlation with both donor and recipient GFR may have partially confounded our study results. Even so, the persistence of an independent association between cystatin C and recipient mGFR even after adjustment for donor age suggests that cystatin C provides valuable information in difficult cases where donor age alone does not fully capture the variability in recipient outcomes.

To account for the effect of body surface area (BSA) in the measurement of GFR, GFR is commonly standardized to ml/min/1.73m^2^. In our study, the association between donor cystatin C and recipient GFR was no longer significant after adjustment for recipient BSA. We hypothesize that this may be explained by the variability introduced when a kidney, of which the function is related to the BSA of the donor is transplanted into a recipient with a different BSA. This problem in judging on kidney function in kidney transplant recipients was already recognized in the setting of living kidney donation [37]. Our results suggest that GFR adjustment for recipient BSA may also not be appropriate in the context of deceased kidney donors.

Our study addresses a crucial gap in current donor evaluation practices by proposing the integration of cystatin C as an additional marker for GFR assessment, particularly where traditional markers like creatinine fall short. A limitation of our study is the use of measurements of donor creatinine and donor cystatin C, which are endogenous filtration markers, rather than assessments of donor kidney function by means mGFR. However, because assessment of mGFR is time-consuming and labor-intensive, direct measurement of donor mGFR is currently not possible in clinical practice. Our study includes a relatively small sample size, highlighting the need for a larger prospective study on biomarkers for graft function in deceased kidney donors to enhance statistical power. Even though we adjusted for multiple potential confounding factors, it cannot be excluded that there are unmeasured confounders, for which we could not adjust. In addition, the included deceased donor population was relatively young and had a low prevalence of hypertension and diabetes, compared to the current extended criteria donor population [38]. However, we expect that the differences between cystatin C and creatinine would then be even more pronounced, because of the larger contribution of older donors with lower muscle-mass in the current extended criteria donor population. Additionally, the availability of the assay and therefore possibility to measure donor cystatin C may be a limiting factor in its introduction to donor screening.

In conclusion, donor plasma creatinine was not associated with recipient mGFR and 24-h creatinine clearance, while donor cystatin C was independently associated with the same outcomes. The latter association remained significant after adjustment for potential confounders These findings suggest that incorporating cystatin C into deceased donor screening may provide additional information in difficult cases and should – be considered as part of the evaluation process of deceased kidney donors.

## Supporting information

S1 Fig STROBE guidelines. (PDF)

S1 Table Associations of donor creatinine, cystatin C with recipient BSA adjusted mGFR (ml/min/1.73 m^2^). There was no missing data in the covariables for the primary analyses. Model 1: adjustment for donor age and sex. Model 2: donor age, sex and donor type. Model 3: donor age, sex and BMI. Model 4: donor age, sex, history of hypertension, smoking and diabetes mellitus. Model 5: donor age, sex and recipient age and sex.
(DOCX)
