# Supplementary material for: Deceased kidney donor cystatin C and subsequent recipient measured glomerular filtration rate at one year after transplantation
Source: PLoS One. 2026 Mar 10;21(3):e0342497. doi: 10.1371/journal.pone.0342497 (PMC12974819; doi:10.1371/journal.pone.0342497)
Supplement: S1 Fig — (PDF) [file pone.0342497.s001.pdf]

STROBE Statement—Checklist of items that should be included in reports of ***cross-sectional studies***

	Item No	Recommendation
**Title and abstract**	1	(*a*) Indicate the study's design with a commonly used term in the title or the abstract<br>**Abstract**
		(*b*) Provide in the abstract an informative and balanced summary of what was done and what was found<br>**Abstract**
**Introduction**		
Background/rationale	2	Explain the scientific background and rationale for the investigation being reported<br>**Introduction paragraph 2 and 3**
Objectives	3	State specific objectives, including any prespecified hypotheses<br>**Introduction paragraph 3**
**Methods**		
Study design	4	Present key elements of study design early in the paper<br>**Methods: "Study Population" and "Data collection"**
Setting	5	Describe the setting, locations, and relevant dates, including periods of recruitment, exposure, follow-up, and data collection<br>**Methods: "Study Population" and "Data collection"**
Participants	6	(*a*) Give the eligibility criteria, and the sources and methods of selection of participants<br>**Methods: "Study Population"**
Variables	7	Clearly define all outcomes, exposures, predictors, potential confounders, and effect modifiers. Give diagnostic criteria, if applicable<br>**Methods: "Statistical Analyses"**
Data sources/ measurement	8*	For each variable of interest, give sources of data and details of methods of assessment (measurement). Describe comparability of assessment methods if there is more than one group<br>**Methods: "Data collection"**
Bias	9	Describe any efforts to address potential sources of bias<br>**Methods: "Statistical Analyses"**
Study size	10	Explain how the study size was arrived at<br>**Methods: "Study Population" and Results: "Figure 1"**
Quantitative variables	11	Explain how quantitative variables were handled in the analyses. If applicable, describe which groupings were chosen and why<br>**Methods: "Statistical Analyses"**
Statistical methods	12	(*a*) Describe all statistical methods, including those used to control for confounding<br>**Methods: "Statistical Analyses"**
		(*b*) Describe any methods used to examine subgroups and interactions<br>**Methods: "Statistical Analyses"**
		(*c*) Explain how missing data were addressed<br>**Methods: "Statistical Analyses"**
		(*d*) If applicable, describe analytical methods taking account of sampling strategy
		(*e*) Describe any sensitivity analyses<br>**Methods: "Statistical Analyses"**
**Results**		
Participants	13*	(a) Report numbers of individuals at each stage of study—eg numbers potentially
