# Supplementary material for: Deceased kidney donor cystatin C and subsequent recipient measured glomerular filtration rate at one year after transplantation
Source: PLoS One. 2026 Mar 10;21(3):e0342497. doi: 10.1371/journal.pone.0342497 (PMC12974819; doi:10.1371/journal.pone.0342497)
Supplement: S1 Table — There was no missing data in the covariables for the primary analyses. Model 1: adjustment for donor age and sex. Model 2: donor age, sex and donor type. Model 3: donor age, sex and BMI. Model 4: donor age, sex, history of hypertension, smoking and diabetes mellitus. Model 5: donor age, sex and recipient age and sex. (DOCX) [file pone.0342497.s002.docx]

**S2 Association of pre-donation creatinine, cystatin C and recipient mGFR and 24-h**

**creatinine clearance**

| ***BSA-adjusted mGFR*** | | | | | | | | |
| --- | --- | --- | --- | --- | --- | --- | --- | --- |
|  | Creatinine | | | | Cystatin C | | | |
|  | **st.ß** | **95% CI** | **R^2^** | **p-value** | **st.ß** | **95% CI** | **R^2^** | **p-value** |
|  | -0.21 | [0.49 to 0.07] | 0.02 | 0.15 | -0.36 | [-0.70 to -0.02] | 0.06 | 0.04 |
| model 1 | -0.10 | [-0.39 to 0.19] | 0.21 | 0.50 | -0.23 | [-0.56 to 0.11] | 0.23 | 0.19 |
| model 2 | -0.10 | [-0.39 to 0.20] | 0.20 | 0.52 | -0.22 | [-0.56 to 0.11] | 0.22 | 0.20 |
| model 3 | -0.11 | [-0.42 to 0.20] | 0.19 | 0.48 | -0.24 | [-0.58 to 0.11] | 0.21 | 0.18 |
| model 4 | -0.14 | [-0.45 to 0.17] | 0.20 | 0.38 | -0.19 | [-0.54 to 0.17] | 0.21 | 0.30 |
| model 5 | -0.12 | [-0.39 to 0.16] | 0.34 | 0.41 | -0.27 | [-0.57 to 0.03] | 0.37 | 0.09 |
| ***BSA-adjusted 24h-creatinine clearance*** | | | | | | | | |
|  | Creatinine | | | | Cystatin C | | | |
|  | -0.26 | [-0.48 to -0.03] | 0.05 | 0.03 | -0.42 | [-0.62 to -0.21] | 0.17 | <0.001 |
| model 1 | -0.30 | [-0.54 to -0.06] | 0.16 | 0.02 | -0.37 | [-0.58 to -0.16] | 0.22 | 0.001 |
| model 2 | -0.31 | [-0.55 to -0.07] | 0.15 | 0.02 | -0.37 | [-0.58 to -0.16] | 0.21 | 0.001 |
| model 3 | -0.30 | [-0.55 to -0.05] | 0.15 | 0.02 | -0.37 | [-0.58 to -0.15] | 0.21 | 0.001 |
| model 4 | -0.33 | [-0.58 to -0.09] | 0.19 | 0.01 | -0.35 | [-0.57 to -0.13] | 0.22 | 0.003 |
| model 5 | -0.30 | [-0.54 to -0.05] | 0.19 | 0.02 | -0.37 | [-0.58 to -0.17] | 0.26 | <0.001 |

Recipient mGFR and 24h-creatinine clearance data were available for 55(69.6%) and 72 (91.1%) recipients, respectively. There was no missing data in the covariables.

Model 1: adjustment for donor age and sex. Model 2: donor age, sex and donor type. Model 3: donor age, sex and BMI. Model 4: donor age, sex, history of hypertension, smoking and diabetes mellitus. Model 5: donor age, sex and recipient age and sex.
